# Endocrine Mucin-Producing Carcinoma: A Case Report With Distinctive Immunohistochemical Features and Correlation With the Literature

**DOI:** 10.7759/cureus.76795

**Published:** 2025-01-02

**Authors:** Gaia Ghiringhelli, Francesco Borelli, Luca Vaienti

**Affiliations:** 1 Plastic and Reconstructive Surgery, Istituto di Ricovero e Cura a Carattere Scientifico (IRCCS) Ospedale Galeazzi-Sant'Ambrogio, Milan, ITA

**Keywords:** empc, endocrine mucin-producing carcinoma, gata3, rare skin tumor, ­skin cancer

## Abstract

This case report describes a rare case of an endocrine mucin-producing carcinoma (EMPC) in an 84-year-old male patient presenting with a cutaneous lesion in the left infraorbital region. EMPC is a rare cutaneous neoplasm characterized by the presence of basaloid cells that produce mucin, alongside features of neuroendocrine differentiation. It often poses diagnostic challenges due to its histological overlap with other skin tumors, such as basal cell carcinoma and mucinous carcinoma. The patient underwent outpatient surgery for the excision of the lesion and reconstruction with a Mustardé-like local flap. Histological examination revealed an ulcerated neoplasm composed of mucin-secreting basaloid cells with focal neuroendocrine differentiation. This case is discussed in the context of the existing literature on EMPC.

## Introduction

Endocrine mucin-producing carcinoma (EMPC) is a rare cutaneous neoplasm characterized by the production of mucin by basaloid cells with endocrine characteristics [1]. It was first described by Flieder and colleagues in 1997 [2]. So far, only a few cases have been reported, and the paucity of literature on the topic often makes its diagnosis challenging. Indeed, EMPC closely resembles other cutaneous neoplasms, such as basal cell carcinoma and squamous cell carcinoma, in its clinical presentation, making it difficult to identify. Additionally, little is known about the most appropriate management of EMPC.

Endocrine mucin-producing sweat gland carcinoma (EMPSGC) is considered part of a morphological continuum with mucinous carcinoma. Although it generally follows an indolent clinical course, EMPSGC is believed to be a precursor of invasive mucinous carcinoma and has the potential for local recurrence [3], as well as for distant metastasis in some cases [4,5].

This neoplasm has a strong predilection for the eyelid, particularly the lower eyelid. Nonetheless, extra-eyelid locations have been also identified [6,7]. EMPSGC predominantly affects the elderly, with a median age of onset around 70 years, ranging from 48 to 84 years. Due to the rarity of EMPSGC, there are no specific data regarding its incidence; however, it appears to occur more frequently in women than in men [7].

Recognized as a distinct entity in the 2018 World Health Organization (WHO) classification of skin tumors, EMPSGC is a low-grade, neuroendocrine-differentiated, cutaneous adnexal tumor [8]. It has histomorphological and immunohistochemical similarities to solid papillary carcinoma of the breast, and both share neuroendocrine features.

Histologically, EMPSGCs appear as well-circumscribed uni- or multi-nodular tumors with solid, cystic, and papillary areas. These tumors typically show a solid growth pattern with small, scattered cysts and a cribriform arrangement, where tumor cells grow in a lace-like network or a pseudo-rosette pattern [6,7]. EMPSGC exhibits a distinctive immunohistochemical profile. Tumor cells typically express CK7, EMA, and neuroendocrine markers such as chromogranin A, synaptophysin, CD56, and neuron-specific enolase (NSE), indicating neuroendocrine differentiation. Furthermore, estrogen receptor (ER) and progesterone receptor (PR) are frequently co-expressed, thus rendering hormone receptor positivity a defining characteristic of this carcinoma. Myoepithelial markers such as calponin, p63, and SMA are useful in the identification of in situ carcinoma components. Tumors are typically negative for CK20, thereby reinforcing their distinct immunophenotype in comparison to other skin neoplasms [3,6,7].

Despite its recognition as a distinct entity, the rarity of EMPSGC makes the diagnosis and management still challenging. The aim of this article is to report a case of this rare cutaneous cancer and to increase knowledge in order to facilitate accurate diagnosis, appropriate treatment, and a more precise prognostic definition for this uncommon tumor.

## Case presentation

An 84-year-old male patient presented to the Plastic Surgery Outpatient Clinic of the Istituto di Ricovero e Cura a Carattere Scientifico (IRCCS) Ospedale Galeazzi-Sant'Ambrogio in Milan in 2024 with a painless cutaneous lesion in the left infraorbital region, with progressive growth and pruritus over the last six months. He had previously been visited by a dermatologist, who recommended excision, suspecting basal cell carcinoma. The patient's medical history includes type 2 diabetes mellitus and a liver malignancy treated with transarterial chemoembolization (TACE).

**Figure 1:**
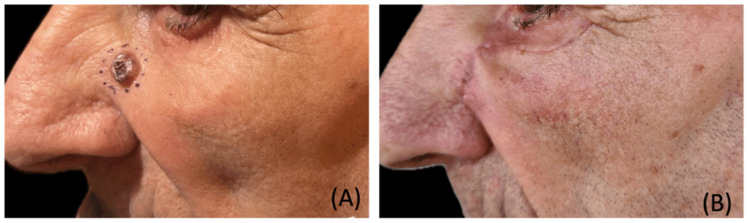
(A) Preoperative image of the infraorbital lesion. (B) Aesthetic result at four weeks postoperatively.

On physical examination, the lesion appeared as an ulcerated nodule, grey in color, approximately 0.8 cm in maximum diameter, with irregular borders and superficial crusting. The lesion was partially ulcerated (Figure 1A).

Surgery was performed on an outpatient basis three months after the initial presentation. The lesion was excised with safety margins, and the resulting defect was reconstructed with a Mustardé-like local flap (Figure 1B). The excised tissue was sent for histopathological examination.

**Figure 2:**
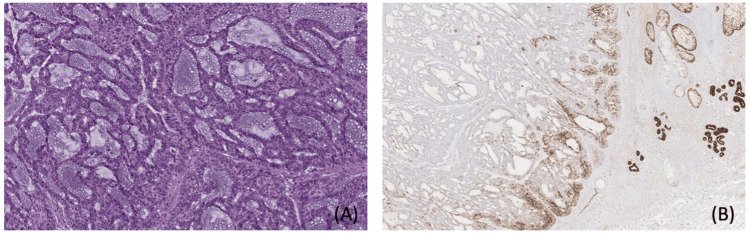
(A) Hematoxylin and eosin-stained section of EMPC, showing basaloid cells with mucin production. (B) Immunohistochemical staining for CK7, highlighting cytoplasmic positivity in tumor cells, confirming epithelial origin. EMPC: endocrine mucin-producing carcinoma

The macroscopic findings described a wedge-shaped piece of skin and subcutaneous tissue measuring 1.5×1.2×1 cm, with an ulcerated greyish neoplasm of 1 cm, located 0.2 cm from the lateral resection margin. Microscopically, the neoplasm was composed of mucin-secreting basaloid cells (Figure 2A). Immunohistochemical analysis showed positivity for p63, p40, CK7, GATA3, and CD56 and focal positivity for chromogranin while being negative for CD117, BerEP4, ER, PR, and synaptophysin (Figure 2B). The SOX10 marker was partially positive. The differential diagnosis included EMPC of eccrine glands and basal cell carcinoma with eccrine differentiation. The resection margins were free of the tumor.

At the four-week postoperative evaluation, the aesthetic and functional results were considered satisfactory. The postoperative period was uneventful. At the six-month follow-up, the patient showed no signs of recurrence or postoperative complications.

## Discussion

Given the rarity of the tumor, the diagnosis of EMPC in our patient was made histologically after excision. Moreover, the diagnosis was challenging due to its unique immunohistochemical characteristics. EMPC typically affects old individuals, with a median age at diagnosis of 70 years, and has a predilection for women [9]. Conversely, our patient was male and significantly older.

Clinically, the lesion appeared with a typical presentation of a single solid, partially ulcerated nodule on the lower eyelid. This led to the initial clinical diagnosis of basal cell carcinoma due to its higher prevalence.

Microscopic analysis revealed basaloid cells secreting mucin, leading to the consideration of EMPC. However, this case presented a diagnostic challenge due to its distinctive immunohistochemical profile. Notably, the tumor was negative for ER and PR, in contrast to the high positivity rates reported in a multicenter retrospective study of 63 cases by Agni et al. [10] where ER and PR positivity rates were 98.2% and 96.1%, respectively.

In our patient, CK7 expression indicated epithelial differentiation, while focal chromogranin positivity suggested neuroendocrine differentiation. However, synaptophysin was negative. Negativity for synaptophysin and focal positivity for chromogranin, although less frequent, have been documented [11].

The positivity for the p63 receptor demonstrated the continuity of the myoepithelium typical of in situ neoplasia.

GATA3 positivity, which is more commonly associated with breast cancer, has only been described rarely in EMPC [12]. In female breast cancer, there is a well-established correlation between GATA3 and ER expression, with GATA3 being predominantly present in ER-positive patients and associated with lower tumor grades. However, studies of GATA3 expression in male breast cancer have yielded inconsistent results in different cohorts [13]. Given the rarity of EMPC, the prognostic significance of different marker expressions is unknown.

In conclusion, our case highlights the diagnostic complexity of EMPC and emphasizes the importance of thorough histopathological evaluation and nuanced interpretation of immunohistochemical markers in distinguishing this rare entity from other cutaneous malignancies. Further studies are warranted to elucidate the clinical significance and prognostic implications of the immunohistochemical profile observed in EMPC, particularly in cases that deviate from the expected demographic and immunophenotypic patterns seen in typical presentations.

## Conclusions

This case of EMPC underlines the diagnostic complexity and the importance of detailed histopathological evaluation in rare cutaneous neoplasms. The clinical presentation and histopathological analysis of our 84-year-old male patient revealed distinctive features that challenged diagnostic expectations given the prevalence of EMPC in elderly women. The immunohistochemical profile in this case deviated from the typical patterns described in the literature, making the diagnosis particularly challenging. Further research efforts are needed to improve our understanding of EMPC, ensure accurate diagnostic criteria, and develop appropriate therapeutic strategies.
